# A scientific research training programme for teaching biomedical students to identify the horizontal transfer of antibiotic resistance genes

**DOI:** 10.1007/s12223-024-01219-3

**Published:** 2024-11-05

**Authors:** Jiafang Fu, Peipei Zhang, Xunzhe Yin, Lingjia Zhu, Gongli Zong, Chuanqing Zhong, Guangxiang Cao

**Affiliations:** 1 Biomedical Sciences College & Shandong Medicinal Biotechnology Centre, Shandong First Medical University & Shandong Academy of Medical Sciences, Ji’nan, China; 2 School of Municipal and Environmental Engineering, Shandong Jianzhu University, Ji’nan, China; 3 Duke Kunshan University, Kunshan, China

**Keywords:** Scientific research training programme, Horizontal gene transfer, Antibiotic resistance gene, Integrative and conjugative element, Biomedical sciences teaching

## Abstract

Worldwide prevalence of multi-antibiotic resistant bacteria is rapidly increasing, and the education of undergraduates and graduates about antibiotic resistance and its associated horizontal gene transfer is critical in the general effort to confront the spread of antibiotic resistance. In this study, a deeper understanding of antibiotic resistance and horizontal gene transfer was achieved by biomedical undergraduate students through a scientific research programme. The enthusiasm of students to participate in the training programme was very high, and results revealed that each student could identify the antibiotic resistance integrative and conjugative element from the *Stenotrophomonas maltophilia* MER1 genome. Each student could also draw the phylogenetic relationship of the antibiotic resistance integrative and conjugative element. In addition, students proved the horizontal transfer of antibiotic resistance genes from *S. maltophilia* MER1 to *Escherichia coli* strain 25DN through conjugation and PCR assays. Each group of students was able to obtain the expected results, indicating that the outcome of the scientific research programme was highly reproducible. This programme improved the theoretical knowledge about antibiotic resistance and horizontal gene transfer and the research skills of biomedical sciences students. Through this programme, students learned that antibiotic resistance genes can be horizontally transferred among different bacteria, laying a solid foundation for students to value the importance of the appropriate use of antibiotics in their future work and life.

**Supplementary Information:**

The online version contains supplementary material available at 10.1007/s12223-024-01219-3.

## Introduction

Worldwide prevalence of multi-antibiotic resistant (MAR) bacteria is rapidly increasing (Bassetti et al. 2018; Kern and Rieg 2020), primarily a result of antibiotic misuse in the medical community, aquaculture, and animal husbandry (Christaki et al. 2020; Mdegela et al. 2021; Pham-Duc et al. 2019). The latest monitoring data of the China antimicrobial resistance surveillance system revealed that the detection rate of MAR bacteria still showed an upward trend (https://www.carss.cn/sys/Htmls/dist/index. html#/resistance). For example, the detection rate of MAR *Klebsiella pneumoniae* showed an increasing trend year by year (from 7.6% in 2015 to 11.3% in 2021), and the detection rate was 26.5% in Shanghai in 2022. The detection rate of MAR bacteria in other countries also revealed an increasing trend, such as a detection rate of 64.1% for MAR bacteria isolated from medical wastewater, as reported in 2022 in Ethiopia (Geta and Kibret 2022). Antibiotic resistance genes (ARGs) from MAR bacteria are considered genetic pollution in the environment (Pruden et al. 2006), and MAR bacteria as well as ARGs from MAR bacteria have become a threat to public health systems globally (Bhardwaj et al. 2022; Frieri et al. 2017). Therefore, the horizontal transfer of ARGs among MAR bacteria is an important topic in both the environmental sciences and medical sciences.

Transformation (Kruger and Stingl 2011), conjugation (Lederberg and Tatum 1946), and transduction (Thierauf et al. 2009) are the three main ways for bacteria to horizontally transfer ARGs. Conjugation is the more common way of horizontal gene transfer (HGT) among bacteria in the environment. A study of 1124 complete prokaryotic genomes identified 180 putative conjugative plasmids and 335 putative integrative and conjugative elements (ICEs) (Guglielmini et al. 2011), suggesting that conjugation via ICEs is likely more common than via conjugative plasmids in prokaryotes. ICEs, which can range from about 20 to 500 kb in size, are mobile genetic elements that are integrated into bacterial chromosomes and that can excise and be transferred to new cells (de Assis et al. 2022). ICEs are integrated into a host genome and encode functional machinery, such as integrons, recombinases, integrases, and type IV secretion/conjugation systems, that enable them to horizontally transfer to other bacteria via conjugation (Christie et al. 2014).

Many students in the biomedical sciences have some level of awareness of antibiotic resistance and understand the need to be cautious when prescribing antibiotics to patients (Sefah et al. 2022; Vazquez-Lago et al. 2023). However, the mechanisms of HGT and the evolution of ARGs are complex, and it is always difficult for students to understand the mechanism of ARGs spread through HGT. Many biological concepts that are central to understanding the spread of MAR bacteria, such as HGT, mobile elements, and the evolution of MAR genes are topics that many students struggle with. In their later years of education, students struggle with intuitive reasoning about biological change being intentional, randomness in biological processes, and the application of physical and chemical concepts in biological processes (Champagne Queloz et al. 2017). Additionally, many biology students think that the transfer of mutated genes is only vertical (Briggs et al. 2017). Richard et al. specifically investigated misconceptions about antibiotic resistance among non-majors, freshmen, and advanced biology majors and found that most students held teleological beliefs about concepts directly related to antibiotic resistance (Richard et al. 2017). Over time, students tend to forget basic scientific knowledge that is not used, revisited, or relearned since it was acquired (Alshamrani et al. 2021; Custers and Ten Cate 2011). Therefore, understanding the spread of MAR pathogenic bacteria and the mechanisms that enable horizontal transfer of ARGs is essential for the training of biomedical undergraduate and graduate students.

Previously published course modules have been designed to either introduce the concepts of resistance genes and HGT or to cover plasmid-mediated conjugation transfer assay procedures in laboratory instructions (Sefah et al. 2022; Vazquez-Lago et al. 2023). It is challenging to help students overcome their misconceptions about antibiotic resistance and horizontal transfer because these misconceptions are widespread and deeply rooted. Using designed active learning to help students overcome their misconceptions has been suggested (Kampourakis 2020). A teaching case study based on the emergence of vancomycin-resistant *Staphylococcus aureus* was used to help students better understand antibiotic resistance and HGT (Boury et al. 2022); however, this teaching case study only took 50 min and mainly focused on theoretical teaching.

Our teaching team teaches biomedical undergraduate and graduate students and also studies the spread of MAR bacteria (Fu et al. 2020, 2021). *Stenotrophomonas maltophilia* MER1 is a MAR bacterium (Xie et al. 2021), and its whole genome has been sequenced; the complete genome sequence of MER1 is available in GenBank under accession no. CP049368. A typical antibiotic resistance ICE (named ICE*Sma*M1) carrying several ARGs was identified in the MER1 genome. In this study, a scientific research programme was used to deepen the understanding of biomedical undergraduate students regarding antibiotic resistance and HGT based on *S. maltophilia* MER1. The framework for the comprehensive scientific research training programme is shown in Figure S1. The learning objectives of the comprehensive scientific research training programme are listed in Table 1.

**Table 1:** Learning objectives of the comprehensive scientific research training programme

Cognition	Skill/Competency	Effectiveness
1. Remember and understand the concepts of antibiotic resistance gene, genomic island, integrative and conjugative element (ICE), and horizontal gene transfer 2. Distinguish between genomic island and ICE 3. Apply analytical methods to identify an antibiotic genomic island and an antibiotic ICE 4. Analyze the horizontal transfer elements in ICE*Sma*M1 5. Evaluate the horizontal transferability of ICE*Sma*M1	1. Cultivate students’ ability to analyze and solve problems 2. Exercise students’ experimental operation skills 3. Improve literature retrieval and writing ability	1. Cultivate students’ learning initiative 2. Stimulate students’ interest in learning 3. Cultivate students’ teamwork and effective communication

## Materials and methods

### Strains used in the scientific research training programme

The MAR *Stenotrophomonas maltophilia* strain MER1 (Xie et al. 2021) was isolated from wastewater and preserved in our laboratory. MER1 was incubated overnight at 28 °C in Luria–Bertani (LB) medium supplemented with 32 mg/L erythromycin. The sodium azide-resistant strain *Escherichia coli* 25DN, which was obtained by mutagenesis and which is sensitive to several antibiotics (Fu et al. 2020, 2021; Li et al. 2019), was grown overnight at 37 °C in LB medium.

### Identification of the antibiotic resistance integrative and conjugative element ICE*Sma*M1

The complete genome sequence of MER1 was obtained from GenBank under accession no. CP049368. IslandViewer 4 (Bertelli et al. 2017) (https://www.pathogenomics.sfu.ca/islandviewer/) was used to analyze genomic islands in the MER1 genome. ICEfinder (Liu et al. 2019) (https://bioinfo-mml.sjtu.edu.cn/ICEfinder/ICEfinder.html) was further used to analyze the ICE (ICE*Sma*M1) in the MER1 genome. Genes in the identified ICE*Sma*M1 were annotated using NCBI and the RASTtk server (Brettin et al. 2015; Overbeek et al. 2014).

### Evolutionary analysis of ICE*Sma*M1

Pairwise alignment of nucleotide sequences of ICE*Sma*M1 with genome sequences of other bacteria was conducted using the BLAST search tool (https://blast.ncbi.nlm.nih.gov/Blast.cgi), and further alignment was conducted using the sequence analysis software BioXM v2.7 (http://202.195.246.60/BioXM/).

### Conjugation assays and verification

To determine if the ARGs in ICE*Sma*M1 could be transferred from *S. maltophilia* MER1 to other bacteria by HGT, conjugation assays were carried out as previously described (Fu et al. 2020). The sodium azide-resistant strain* E. coli* 25DN was used as the recipient strain, and *S. maltophilia* MER1 was used as the donor strain. The macrolide ARGs *macA* and *macB* encode a macrolide-specific ABC-type efflux carrier and mediate erythromycin resistance (Kobayashi et al. 2001; Li et al. 2023), and *macA* and *macB* genes were annotated in ICE*Sma*M1; therefore, the macrolide antibiotic erythromycin was used to screen the transconjugants. MER1 (donor) and *E. coli* 25DN (recipient) are inhibited by sodium azide and erythromycin, respectively, and only the transconjugants of *E. coli* can survive on the selective medium and degrade 5-bromo-4-chloro-3-indolyl-β-D-glucuronic acid (X-Gluc) to blue compounds. MER1 and 25DN were grown to the logarithmic growth phase and centrifuged at 5000 rpm to collect the bacterial sediment. The sediments were washed three times using LB medium, and then, the bacterial sediments were resuspended in LB medium. Equal amounts of MER1 and 25DN were mixed with LB broth and incubated at 37 °C with shaking at 220 rpm for 30 min and then incubated at 37 °C for 3 h. Finally, the mixture was spread and cultured on LB agar plates with erythromycin (32 mg/L), sodium azide (3.4 mmol/L), and X-Gluc (100 mg/L), at 37 °C for 36 h; transconjugants were observed as blue colonies. Equal amounts of sterilized water and the 25DN mixture were used for the negative control. The presence of the 5 kb DNA fragment that included the two ARGs *macA* and *macB* of ICE*Sma*M1 was demonstrated in the transconjugants by polymerase chain reaction (PCR) using primers (ICE-VerF: GCGGGCTATGTCAGTTTTGC and ICE-VerR: GCAGCAGGAACTGGACAATC) with visualization by 1% agarose gel electrophoresis.

### Implementation and evaluation of the scientific research programme

The training programme was implemented in the microbial pharmacology course and was conducted among 42 third-year undergraduate students and 30 first-year graduate students in the biomedical sciences. The 42 third-year undergraduate students were 21 to 22 years old, and the male to female ratio was approximately 1:1. The 30 first-year graduate students were 22 to 24 years old, and the male to female ratio was about 2:3. The third-year undergraduate students had taken theoretical courses in microbiology, cytobiology, biochemistry, and molecular biology and taken the corresponding experimental courses. The first-year graduate students had also taken theoretical courses, such as biochemistry, molecular biology, microbiology, bioinformatics, and genetics, and taken the corresponding experimental/practical courses. To assess the students’ knowledge of MAR, ARGs, and HGT and to determine what the instructors should focus on during the implementation of the programme, a pre-test (Table S1) was firstly performed. Detailed descriptions of implementation activities that were part of the scientific research programme are shown in Table S2.

We designed the assessment content according to the learning objectives listed in Table 1. As an evaluation, each student participating in this training programme was asked to write four practical training reports. The four reports and critical points of each report have been summarized in Table 2. The four practical training reports were scored by four instructors, and the rubrics and criteria used to assign scores to the students’ practical training reports were designed for this study (Table 3). Each practical training report was evaluated on a scale with five levels: excellent, good, moderate, pass, and poor, which were assigned scores of 90–100, 80–89, 70–79, 60–69, and less than 60, respectively.

**Table 2:** The critical points of each report

Report	The critical point
Analysis of ICE*Sma*M1 identification	The position of ICE*Sma*M1 in the MER1 genome Antibiotic resistance genes in ICE*Sma*M1 Genetic elements in ICE*Sma*M1 Visualize the structure of ICE*Sma*M1 in a figure Bioinformatics analysis process Valuable conclusions
Description of ICE*Sma*M1 phylogenetic relationship	Analyzing the identities between genes in ICE*Sma*M1 and other bacterial genomes Visualize the phylogenetic relationship of ICE*Sma*M1 in a figure Bioinformatics analysis process Valuable conclusions
Description of the ICE*Sma*M1 conjugation transfer and verification	Present results of conjugation assay by figure Present results of PCR assay by figure Valuable conclusions
Explanations for the ICE*Sma*M1 horizontal transfer	Mechanism of horizontal gene transfer Explanations of horizontal transfer of ICE*Sma*M1 to *E. coli* 25DN Valuable conclusions

**Table 3:** The rubrics and criteria of the students’ practical training reports

Rubric	Criteria	Score
Basic knowledge	The evaluation is made according to the comprehensiveness, accuracy, logic, and profundity of core concepts (antibiotic resistance gene, genomic island, integrative and conjugative element, and horizontal gene transfer), basic principle of horizontal gene transfer. (30 points)	27 ≤ Excellent ≤ 30
		24 ≤ Good < 27
		21 ≤ Moderate < 24
		18 ≤ Pass < 21
		Poor < 18
Analyzing and solving problems	Correct processing of bioinformatics analysis for identifying an antibiotic genomic island and an antibiotic integrative and conjugative element; correct processing of experimental data of conjugation and PCR assays; draw valuable conclusions. (30 points)	27 ≤ Excellent ≤ 30
		24 ≤ Good < 27
		21 ≤ Moderate < 24
		18 ≤ Pass < 21
		Poor < 18
Innovation of report	Pay attention to the latest horizontal gene transfer knowledge and have a sense of innovation in the process of writing reports: improved analytical method or have unique insights. (20 points)	18 ≤ Excellent ≤ 20
		16 ≤ Good < 18
		14 ≤ Moderate < 16
		12 ≤ Pass < 14
		Poor < 12
Quality of writing	The narrative is concise and complete; conclusions are rigorous and reasonable. The characters are smooth, the technical terms are accurate, the symbols are unified, the literature citations are standardized, and the charts are complete. (20 points)	18 ≤ Excellent ≤ 20
		16 ≤ Good < 18
		14 ≤ Moderate < 16
		12 ≤ Pass < 14
		Poor < 12

### Assessment of skills improved through the scientific research training programme

The generic skills and research skills that the students improved upon through the training programme were assessed. The generic skills included learning initiative and effective communication skills; the research skills included literature searching ability, bioinformatics analytical ability, experimental operation skills, scientific report writing ability, the ability to analyze and summarize, and the ability to identify antibiotic resistance ICEs from a microbial genome. Assessment of skills that were improved through the training programme was conducted by means of an online questionnaire (Ge et al. 2022). According to the learning objectives of the scientific research training programme, the online questionnaire was designed as shown in Table S3. The categories of basic knowledge of HGT, research skills, and generic skills in the questionnaire addressed the ‘cognition’, ‘competency’, and ‘effectiveness’ learning objectives, respectively (Table 1). Understanding and mastery of basic knowledge about the horizontal transfer of ARGs were also surveyed by the online questionnaire, which was completed anonymously by the students in the training programme. The assessment consisted of nine items, each of which could be answered as previously described (Dourado et al. 2021; Ge et al. 2022): strongly disagree, rather disagree, neutral, rather agree, and strongly agree. The questionnaire took each student approximately 5 min to complete and was not mandatory for successful completion of the training programme.

### Statistical analysis

The scores of the four reports (listed in Table 2) from the undergraduate students were compared respectively with those of the graduate students. The comparison between groups was conducted by *t*-test. Statistical analysis was analyzed using GraphPad Prism 8.0 for Windows. *p* < 0.05 was considered statistically significant.

## Results

### Antibiotic resistance-conferring ICE*Sma*M1

**Fig. 1:**
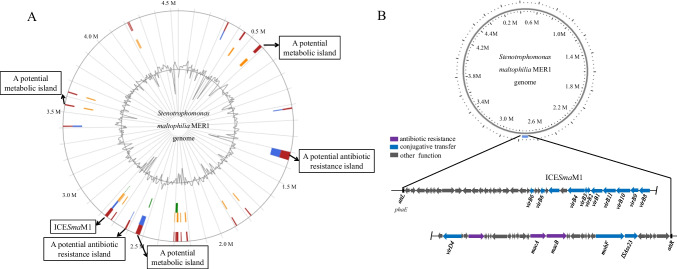
Schematic view of genomic islands and the antibiotic resistance-conferring ICE*Sma*M1 in the MER1 genome. **A** Students analyzed the genomic islands using IslandViewer 4. **B** Students analyzed integrative and conjugative elements using ICEfinder and performed a schematic view analysis. The analysis results of one group of students are presented

Genomic islands are typically characterized as discrete DNA segments that reside on the chromosome. Some genomic islands are mobile whereas others are not or are no longer mobile (Hacker and Carniel 2001); ICE is one type of mobile genomic island. There are several genomic islands in the MER1 genome (Fig. 1A). However, only one genomic island is identified as an antibiotic resistance ICE (named ICE*Sma*M1, Fig. 1B). Genes annotated in ICE*Sma*M1 are listed in Table S4.

Seventy-two students in total participated in this scientific research programme, and these 72 students were divided into 24 groups of three students per group. The analysis results from one group of students are presented in Fig. 1. A comparison of the ICE*Sma*M1 analysis among the 24 groups is shown in Table S5.

### Training in determining the phylogenetic relationship of ICE*Sma*M1

**Fig. 2:**
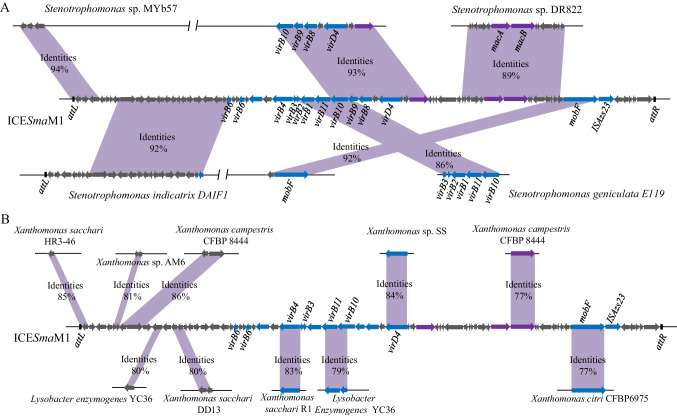
Schematic showing phylogenetic relationship of genes in ICE*Sma*M1. The analysis results of two groups of students are presented. **A** Comparison of nucleotide sequences of ICE*Sma*M1 with genome sequences of *Stenotrophomonas* strains. **B** Comparison of nucleotide sequences of ICE*Sma*M1 with genome sequences of all bacteria excluding *Stenotrophomonas*

The phylogenetic relationship of ICE*Sma*M1, as determined by two groups of students, is presented in Fig. 2. One group of students determined that ICE*Sma*M1 mainly evolved from *Stenotrophomonas* species (Fig. 2A), while the other group of students excluded the *Stenotrophomonas* and found that the antibiotic resistance gene *macB* in ICE*Sma*M1 may have evolved from the *Xanthomonas campestris* CFBP 8444 genome, as the ICE*Sma*M1 *macB* gene had 77% identity to a homologue in that genome (Fig. 2B). Moreover, in the opinion of the second group of students, ICE*Sma*M1 may also have evolved from *Xanthomonas* and *Lysobacter* species (Fig. 2B). A comparison of the ICE*Sma*M1 phylogenetic relationship analyses conducted by the 24 groups is shown in Table S6.

### Visualization of ICE*Sma*M1 horizontal transfer

**Fig. 3:**
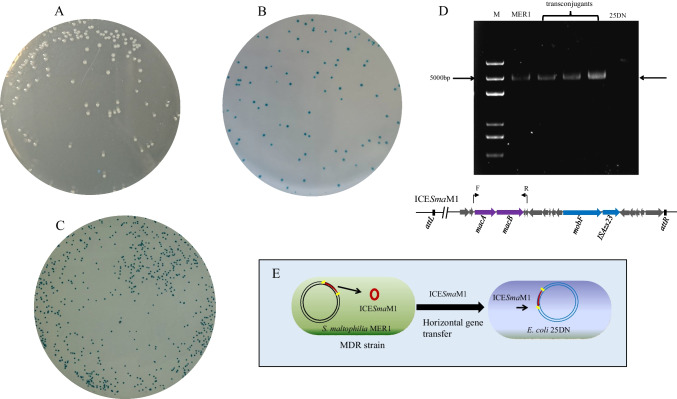
Visualization of ICE*Sma*M1 horizontal transfer from strain MER1 to strain 25DN. **A** *S. maltophilia* MER1 on Luria–Bertani (LB) agar plates with 32 mg/L erythromycin. **B** *E. coli* 25DN on LB agar plates with 3.4 mmol/L sodium azide and 100 mg/L 5-bromo-4-chloro-3-indolyl-β-D-glucuronic acid. **C** The transconjugants on LB agar plates with 32 mg/L erythromycin, 3.4 mmol/L sodium azide, and 100 mg/L 5-bromo-4-chloro-3-indolyl-β-D-glucuronic acid. **D** ICE*Sma*M1 regions were amplified by PCR using the following templates: total DNA of MER1, total DNA of transconjugants, and total DNA of 25DN. M, *Trans*8K DNA marker. **E** A simple model drawn by one group of students showing the horizontal gene transfer of ICE*Sma*M1

Each group of students could visualize the horizontal transfer of ARGs from *S. maltophilia* MER1 to *E. coli* strain 25DN through conjugation and PCR assays. The conjugation and PCR results of one group of students are presented in Fig. 3. MER1 was able to grow in the presence of erythromycin (Fig. 3A), and the sodium azide-resistant *E. coli* 25DN was able to grow in the presence of sodium azide and to degrade the X-Gluc to blue compounds (Fig. 3B). However, only the transconjugants could grow in the presence of both sodium azide and erythromycin and degrade X-Gluc to blue compounds (Fig. 3C). Many blue transconjugants grew after incubation (Fig. 3C), and then, several blue colonies were randomly selected for PCR verification (Fig. 3D). A 5-kb ICE*Sma*M1 region was detected in the transconjugants (Fig. 3D), confirming that ICE*Sma*M1 had been transferred to *E. coli* 25DN. A simple model was drawn by one group of students showing that ICE*Sma*M1 horizontally transferred from the *S. maltophilia* MER1 strain to the *E. coli* 25DN strain (Fig. 3E), suggesting that students grasped the learning point of HGT.

### Assessment of the effectiveness of the comprehensive scientific research training

**Fig. 4:**
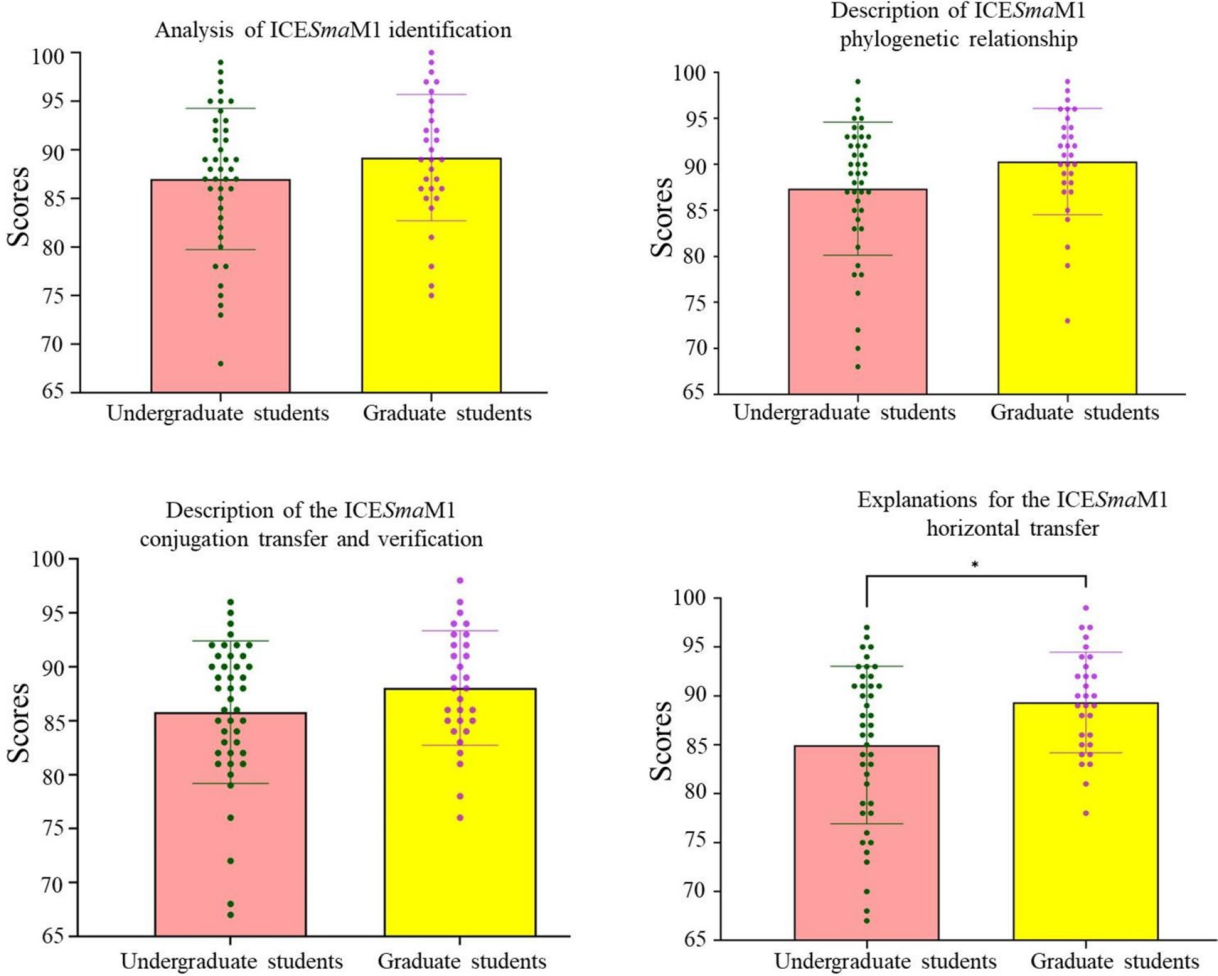
The comprehensive scientific research training effect assessment. As an assessment, each of the 72 students in this training programme was asked to write four practical training reports. Each report was evaluated on a scale with five levels: excellent, good, moderate, pass, and poor, which were assigned scores of 90–100, 80–89, 70–79, 60–69, and less than 60, respectively. Data are expressed as mean ± SD; *p < 0.05

As shown in Fig. 4, both undergraduate and graduate students scored an average of over 85 for each item on the assessment. The numbers of students with an excellent, good, moderate, pass, and poor score are shown in Table S7. For assessment of the analysis of ICE*Sma*M1 identification, description of the ICE*Sma*M1 phylogenetic relationship, and description of the ICE*Sma*M1 conjugation transfer and verification, the scores of undergraduate students were slightly lower than those of graduate students, but there was no significant difference. However, for the assessment of explanations for the horizontal transfer of ICE*Sma*M1, the scores of undergraduate students were significantly lower than those of graduate students (*p* < 0.05), indicating that there are differences in the depth and breadth of knowledge among the third-year undergraduate students and first-year graduate students.

### Scientific research literacy of students improved upon successful completion of the training

**Fig. 5:**
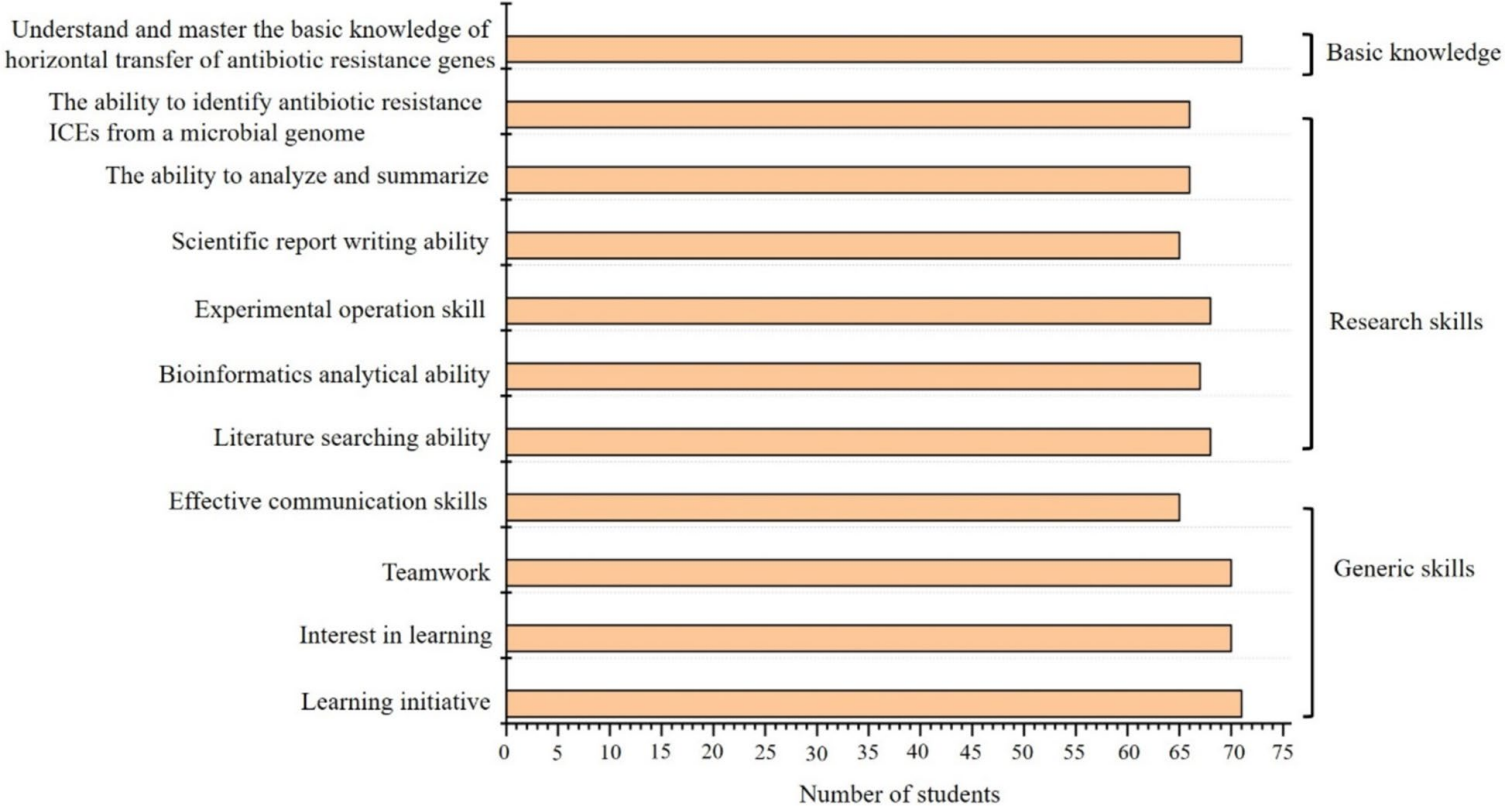
Generic skills and research skills of students were improved through the scientific research training programme. The improvement of students’ generic skills and research skills was evaluated by means of a questionnaire among all of the 72 students. The number of students who strongly agreed is presented

All 72 students who participated in the scientific research training programme submitted questionnaires. Figure 5 summarizes the survey responses and shows that almost all students (71 students) strongly agreed that they had understood and mastered the basic knowledge of the horizontal transfer of ARGs through this training. More than over 90% of the students strongly agreed that their generic skills and research skills had improved through this training. Seventy-one students strongly agreed that their learning initiative improved; 70 students strongly agreed that their interest in learning and teamwork skills improved, and 65 students strongly felt that both their effective communication skills and their ability to write scientific reports had improved. Additionally, 68 students strongly felt that both their ability to search the literature and their experimental operation skill had improved; 67 students strongly felt that they had improved their bioinformatics analytical ability, and 67 students strongly felt that both their ability to analyze and summarize and their ability to identify antibiotic ICEs from a microbial genome had improved.

## Discussion

MAR bacteria and antibiotic resistance are a global concern. However, while many students are aware of this issue, many of them are unclear on the mechanisms by which ARGs may emerge and spread. Many students believe that a mutated gene can only be vertically transferred in organisms (Briggs et al. 2017), and many students are not familiar with the mechanism of HGT (Boury et al. 2022). Here, through a pre-test, we similarly found that students had a very shallow understanding of core concepts of HGT and that many students are only familiar with plasmids but not with genomic islands and ICEs. We also found that the students did not understand how ARGs spread or understand the mechanism of HGT. Boury et al. developed 50-min-long ‘interrupted case studies’ to help students understand HGT (Boury et al. 2022). However, those case studies were limited to theoretical teaching, and students could not experimentally instead of effectively analyze and verify the horizontal transfer of ARGs in the short time given for the studies.

In this study, we first describe a comprehensive training programme for teaching biomedical students to identify and visualize the horizontal transfer of ARGs. Based on conjugation between *S. maltophilia* MER1 and *E. coli* 25DN, this programme helped students to understand the mechanisms by which ARGs may spread. Through analyzing and identifying the antibiotic resistance-conferring ICE*Sma*M1 from the *S. maltophilia* MER1 genome, students better understood how ARGs can spread and evolve. Data in Fig. 1 and Table S5 revealed that students had mastered how to identify and annotate an antibiotic resistance ICE in a microbial genome. Data in Fig. 2 and Table S6 revealed that students were able to proactively propose analysis strategies, indicating that students were very interested in the project and had mastered the analysis methods. All 24 groups of students screened transconjugants on the screening plates, and the number of transconjugants obtained varied between groups. However, all groups detected the transfer of ICE*Sma*M1 to *E. coli* 25DN, indicating the conjugation assays and PCR verification assays were highly reproducible. Additionally, the students’ understanding of the horizontal transfer of ARGs mediated by ICEs was strengthened through performing the conjugation assays.

The 50-min ‘interrupted case studies’ approach is appropriate for small and large groups of students (Boury et al. 2022). However, a teaching programme that involves experimentation is better suited to small groups. Ge et al. developed a positron emission tomography imaging training programme for small groups of students (40 students) to help the students understand certain pharmacological parameters and concepts of positron emission tomography, and this programme was found to be an effective teaching method (Ge et al. 2022). Our study revealed that our training programme was also effective. For this training programme, more than 65 students strongly agreed that their generic skills and research skills had been improved, and almost all students strongly agreed that they had mastered the basic knowledge of the horizontal transfer of ARGs. This training programme greatly improved students’ interest in learning and their scientific research literacy, and students themselves generally believed that they had a deeper understanding of MAR bacteria, ARGs, and the evolution and horizontal transfer of ARGs.

Most ARGs are found on ICEs and primarily spread by conjugation (Fu et al. 2023; Guglielmini et al. 2011). ICEs act as a reservoir to maintain antibiotic resistance in the bacterial population in the absence of antibiotic selection. However, conjugation via ICE can spread ARGs and antibiotic resistance quickly among bacteria of the microbiome and pathogens under the pressure of antibiotics. A conjugation inhibitor that blocks the conjugation process but does not affect cell growth was able to prevent the spread of antibiotic resistance (Graf et al. 2019). Our programme enabled students to master the basic characteristics of the ICE and the elements required for HGT through the exploration of one antibiotic ICE, namely ICE*Sma*M1. This programme further cultivated students’ ability to analyze other antibiotic ICEs and other categories of ICES, such as metabolic, virulence, adaptive, ecological, symbiotic, and saprophytic ICEs. The ability of students to further determine whether these ICEs can be horizontally transferred to other strains was also enhanced through this programme. In addition, through this training programme, students realized that the rational use of antibiotics and a conjugation inhibitor are effective ways to inhibit the spread of ARGs among bacteria in the environment.

## Conclusion

The results of our scientific research programme revealed that each participating student could identify the antibiotic resistance-conferring ICE*Sma*M1 from the *S. maltophilia* MER1 genome, and each student could draw the phylogenetic relationship of ICE*Sma*M1. In addition, each group of students visualized the horizontal transfer of ARGs from *S. maltophilia* MER1 to *E. coli* strain 25DN through conjugation and PCR assays. This programme improved the theoretical knowledge of antibiotic resistance and HGT and the research skills of biomedical sciences students. Almost all students strongly agreed that they had understood and mastered the basic knowledge of the horizontal transfer of ARGs through this training. Hence, this comprehensive scientific research training programme can be applied to the teaching of students in medicine, life and health sciences, biology, pharmacy, environmental science, and other areas.

## Supplementary Information

Below is the link to the electronic supplementary material.

Supplementary file1 (DOCX 260 KB)

## Data Availability

This study does not involve data that need to be deposited into a publicly available repository. All data have been included in the article and supplementary materials.
