# Supplementary material for: A scientific research training programme for teaching biomedical students to identify the horizontal transfer of antibiotic resistance genes
Source: Folia Microbiol (Praha). 2024 Nov 5;70(4):811–21. doi: 10.1007/s12223-024-01219-3 (PMC12476391; doi:10.1007/s12223-024-01219-3)
Supplement: Supplementary file 1 — Supplementary file1 (DOCX 260 KB) [file 12223_2024_1219_MOESM1_ESM.docx]

**Supplementary Materials**

**A** **scientific research training programme for teaching biomedical students to identify the horizontal transfer of antibiotic resistance genes**

Jiafang Fu^1^, Peipei Zhang^1^, Xunzhe Yin^2^, Lingjia Zhu^3^, Gongli Zong^1^, Chuanqing Zhong^2*^, Guangxiang Cao^1*^

^1^Biomedical Sciences College & Shandong Medicinal Biotechnology Centre, Shandong First Medical University & Shandong Academy of Medical Sciences, Ji’nan, China

^2^School of Municipal and Environmental Engineering, Shandong Jianzhu University, Ji’nan, China

^3^Duke Kunshan University, Kunshan, China

***** Corresponding author

Chuanqing Zhong, E-mail address: zhongchuanqing@sdjzu.edu.cn.

Guangxiang Cao, E-mail address: caoguangxiang@sdfmu.edu.cn.

Running title: Training programme for biomedical students

**Table S1**. **The content of pre-test used to evaluate students participated in the scientific research training programme.**

| Content |
| --- |
| What are multi-antibiotic resistant bacteria? |
| What are antibiotic resistance genes? |
| What is a plasmid? Please explain it. |
| What is a genomic island? Please explain it. |
| What is an integrative and conjugative element? Please explain it. |
| How do you understand horizontal gene transfer? |
| Can a plasmid transfer from one strain to another strain? |
| Can gene clusters located on chromosomes be transferred from one strain to another? |
| Do you think antibiotic resistance genes can spread among bacteria? How is it spread? |

**Table S2**. **Descriptions of activities involved in the scientific research training programme for biomedical students.**

| Activity | Activity description | Activity type |
| --- | --- | --- |
| Identification of the antibiotic resistance integrative and conjugative element ICE*Sma*M1 | Theoretical knowledge related to antibiotic resistance and horizontal gene transfer such as concepts of multi-antibiotic resistant, multi-antibiotic resistant gene, genomic island, integrative and conjugative element was firstly reviewed. Then, the students are trained to analyze genomic island using IslandViewer 4, analyze integrative and conjugative element using ICEfinder, and annotate genes using NCBI and the RASTtk server. Finally, students worked in groups of three; under the guidance of their lead instructors, each group obtained the MER1 genome data through NCBI, analyzed the genomic island using IslandViewer 4, analyzed integrative and conjugative element using ICEfinder, annotate genes using NCBI and the RASTtk server, and each group was asked to characterize genomic island and integrative and conjugative element to the rest of the class. | Bioinformatics analysis |
| Evolutionary analysis of ICE*Sma*M1 | Theoretical knowledge related to evolution was firstly reviewed. Then, the students are trained to use the BLAST search tool and analyze gene sequences using BioXM v2.7. Finally, students worked in groups of three; under the guidance of their lead instructors, each group analyzed the evolutionary relationship of ICE*Sma*M1 usng the BLAST search tool and BioXM v2.7. Each group was asked to present the evolutionary relationship of ICE*Sma*M1 to the rest of the class. | Bioinformatics analysis |
| Conjugation assays and verification | Theoretical knowledge related to conjugation transfer, PCR, agarose gel electrophoresis was firstly reviewed. Then, the experimental process and its precautions in detail were introduced to students. Finally, students worked in groups of three; underthe guidance of their lead instructors, each group needs to perform the conjugation assays, PCR, and agarose gel electrophoresis assays. Students were required to observe their results carefully and record them accurately. | Experimental operation |
| Evaluation of the scientific research training effect | Each student participating in this training programme was asked to write four practical training reports. | Creative Writing |
| Assessment of skills improved through the scientific research training programme | The students were organized to fill out the online questionnaire. The online questionnaire took each student approximately five minutes to complete. | Online questionnaire |

**Table S3**. **A questionnaire used to assess the scientific research training programme.**

|  | Item | strongly disagree | rather disagree | neutral | rather agree | strongly agree |
| --- | --- | --- | --- | --- | --- | --- |
| Basic knowledge of horizontal gene transfer | Deepened understanding of multi-antibiotic resistant bacteria and multi-antibiotic resistant genes |  |  |  |  |  |
|  | Deepened understanding of evolution |  |  |  |  |  |
|  | Improved understanding of genomic island and integrative and conjugative element |  |  |  |  |  |
|  | Improved understanding of horizontal transfer elements |  |  |  |  |  |
|  | Deepened understanding of horizontal gene transfer |  |  |  |  |  |
|  | Mastered the key points of PCR technology |  |  |  |  |  |
|  | Understand the principles and procedures of conjugation assays |  |  |  |  |  |
|  | Understand the principles and procedures of agarose gel electrophoresis |  |  |  |  |  |
|  | Satisfied with this scientific research training programme |  |  |  |  |  |
| Research skills | Improved ability to identify a genomic island from a microbial genome |  |  |  |  |  |
|  | Improved ability to identify an integrative and conjugative element from a microbial genome |  |  |  |  |  |
|  | Improved ability to analyze evolution of a genomic island or an integrative and conjugative element |  |  |  |  |  |
|  | Improved ability to analyze and summarize |  |  |  |  |  |
|  | Improved scientific report writing ability |  |  |  |  |  |
|  | Improved ability of experimental operations |  |  |  |  |  |
|  | Greatly improved ability of bioinformatics analysis |  |  |  |  |  |
|  | Improved literature retrieval ability |  |  |  |  |  |
| Generic skills | Greatly improved the learning enthusiasm and learning initiative |  |  |  |  |  |
|  | Greatly cultivated the interest in learning and scientific research |  |  |  |  |  |
|  | Improved the ability to communicate with classmates |  |  |  |  |  |
|  | Improved the ability to cooperate with team members |  |  |  |  |  |

**Table S4. Genes annotated in ICE*Sma*M1.**

| start | end | strand | gene | name |
| --- | --- | --- | --- | --- |
| 2699796 | 2700854 | - | *phaE* | class III poly(R)-hydroxyalkanoic acid synthase subunit PhaE |
| 2700877 | 2701491 | - |  | phosphatidylcholine/phosphatidylserine synthase |
| 2701837 | 2702484 | + |  | class I SAM-dependent methyltransferase |
| 2702586 | 2703956 | - |  | MATE family efflux transporter |
| 2704072 | 2704698 | - |  | DUF2239 family protein |
| 2704861 | 2705349 | - |  | 8-oxo-dGTP diphosphatase |
| 2705390 | 2705902 | - |  | DUF1249 domain-containing protein |
| 2706126 | 2706947 | - |  | kinase/pyrophosphorylase |
| 2707093 | 2709471 | + | *ppsA* | phosphoenolpyruvate synthase |
| 2709623 | 2710516 | + |  | mechanosensitive ion channel |
| 2710595 | 2711719 | - |  | alkene reductase |
| 2711908 | 2712780 | - |  | EcsC family protein |
| 2712875 | 2713447 | - | *orn* | oligoribonuclease |
| 2713523 | 2714047 | - | *tadA* | tRNA adenosine(34) deaminase TadA |
| 2714206 | 2714643 | + |  | acetyltransferase |
| 2714674 | 2715186 | - |  | N-acetyltransferase |
| 2715579 | 2716166 | + |  | hypothetical protein |
| 2716163 | 2717482 | + |  | lipase |
| 2717545 | 2718486 | - |  | hypothetical protein |
| 2719074 | 2720090 | - |  | lytic enzyme |
| 2720148 | 2720684 | - |  | hypothetical protein |
| 2721313 | 2721709 | - | *virB6* | type IV secretion system protein |
| 2721784 | 2722314 | - |  | DUF4189 domain-containing protein |
| 2723234 | 2723692 | - |  | DUF4189 domain-containing protein |
| 2723799 | 2724428 | - | *virB6* | type IV secretion system protein |
| 2724588 | 2725064 | - |  | DUF4189 domain-containing protein |
| 2725158 | 2726297 | - |  | type IV secretion system protein |
| 2726373 | 2727203 | - |  | hypothetical protein |
| 2727226 | 2729667 | - | *virB4* | VirB4 family type IV secretion/conjugal transfer ATPase |
| 2729752 | 2730060 | - | *virB3* | VirB3 family type IV secretion system protein |
| 2730053 | 2730439 | - | *virB2* | TrbC/VirB2 family protein |
| 2730564 | 2731544 | - | *virB1* | transglycosylase SLT domain-containing protein |
| 2731548 | 2732591 | - | *virB11* | P-type DNA transfer ATPase VirB11 |
| 2732604 | 2733878 | - | *virB10* | TrbI/VirB10 family protein |
| 2733875 | 2734654 | - | *virB9* | TrbG/VirB9 family P-type conjugative transfer protein |
| 2734651 | 2735697 | - | *virB8* | type IV secretion system protein |
| 2735787 | 2736233 | - |  | hypothetical protein |
| 2736650 | 2738329 | - | *virD4* | Coupling protein VirD4, ATPase required for T-DNA transfer |
| 2738367 | 2738624 | - |  | hypothetical protein |
| 2738747 | 2739166 | + |  | phosphohydrolase |
| 2739277 | 2740488 | + |  | MFS transporter |
| 2740636 | 2741109 | + |  | hypothetical protein |
| 2741222 | 2741491 | + |  | hypothetical protein |
| 2741621 | 2741869 | + |  | hypothetical protein |
| 2741908 | 2743443 | - |  | FAD-dependent monooxygenase |
| 2743526 | 2744230 | + | *tetR* | TetR/AcrR family transcriptional regulator |
| 2744242 | 2744649 | - |  | hypothetical protein |
| 2744870 | 2745151 | - |  | hypothetical protein |
| 2745361 | 2746035 | + |  | response regulator transcription factor |
| 2746053 | 2747384 | + |  | HAMP domain-containing histidine kinase |
| 2747706 | 2748128 | + |  | hypothetical protein |
| 2748265 | 2749455 | + | *macA* | Macrolide-specific efflux protein MacA |
| 2749452 | 2751416 | + | *macB* | Macrolide export ATP-binding/permease protein MacB |
| 2751507 | 2751899 | + |  | hypothetical protein |
| 2751927 | 2752310 | + |  | EF-hand domain-containing protein |
| 2752359 | 2753684 | - |  | hemolysin |
| 2753711 | 2754574 | - |  | hypothetical protein |
| 2755095 | 2755454 | + |  | helix-turn-helix domain-containing protein |
| 2755528 | 2755935 | - |  | hypothetical protein |
| 2756425 | 2757159 | - |  | hypothetical protein |
| 2757352 | 2760105 | + | *mobF* | MobF family relaxase |
| 2760391 | 2761638 | + | *ISAzs23* | integrase |
| 2761596 | 2762564 | - |  | hypothetical protein |
| 2762705 | 2763205 | - |  | DUF4365 domain-containing protein |
| 2763303 | 2763788 | - |  | hypothetical protein |
| 2763877 | 2764251 | - |  | BLUF domain-containing protein |
| 2764995 | 2766680 | + |  | hypothetical protein |

**Table S5. Comparison of ICE*Sma*M1 analysis among 24 groups.**

|  | Group | Accurate analysis of genomic islands | Accurate analysis of ICE*Sma*M1 position | Accurate analysis of antibiotic resistance genes in ICE*Sma*M1 | Accurate analysis of genetic elements in ICE*Sma*M1 | Correct draw the ICE*Sma*M1 Structure |
| --- | --- | --- | --- | --- | --- | --- |
| Undergraduate students | 1 | Yes | Yes | Yes | Yes | Yes |
|  | 2 | Yes | Yes | Yes | Yes | Yes |
|  | 3 | Yes | Yes | basically correct | Yes | basically correct |
|  | 4 | Yes | Yes | Yes | Yes | Yes |
|  | 5 | Yes | Yes | Yes | Yes | Yes |
|  | 6 | Yes | Yes | Yes | Yes | Yes |
|  | 7 | Yes | Yes | basically correct | Yes | basically correct |
|  | 8 | Yes | Yes | Yes | Yes | Yes |
|  | 9 | Yes | Yes | Yes | Yes | Yes |
|  | 10 | Yes | Yes | Yes | Yes | Yes |
|  | 11 | Yes | Yes | Yes | basically correct | basically correct |
|  | 12 | Yes | Yes | Yes | Yes | Yes |
|  | 13 | Yes | Yes | Yes | Yes | Yes |
|  | 14 | Yes | Yes | Yes | basically correct | basically correct |
| Graduate students | 15 | Yes | Yes | Yes | Yes | Yes |
|  | 16 | Yes | Yes | Yes | Yes | Yes |
|  | 17 | Yes | Yes | Yes | Yes | Yes |
|  | 18 | Yes | Yes | Yes | Yes | Yes |
|  | 19 | Yes | Yes | Yes | basically correct | basically correct |
|  | 20 | Yes | Yes | Yes | Yes | Yes |
|  | 21 | Yes | Yes | Yes | Yes | Yes |
|  | 22 | Yes | Yes | Yes | Yes | Yes |
|  | 23 | Yes | Yes | Yes | Yes | Yes |
|  | 24 | Yes | Yes | Yes | Yes | Yes |

**Table S6. Comparison of ICE*Sma*M1 phylogenetic relationship analysis among 24 groups.**

|  | Group | Comparison ICE*Sma*M1 with all organisms excluding *Stenotrophomonas* | Comparison ICE*Sma*M1 with *Stenotrophomonas* | Accurate analysis of identities between different DNA fragments | Correct draw the phylogenetic relationship of ICE*Sma*M1 |
| --- | --- | --- | --- | --- | --- |
| Undergraduate students | 1 | / | Yes | Yes | Yes |
|  | 2 | Yes | Yes | Yes | Yes |
|  | 3 | Yes | / | Yes | Yes |
|  | 4 | / | Yes | Yes | basically correct |
|  | 5 | Yes | / | Yes | Yes |
|  | 6 | / | Yes | Yes | Yes |
|  | 7 | Yes | / | Yes | Yes |
|  | 8 | / | Yes | Yes | Yes |
|  | 9 | / | Yes | Yes | Yes |
|  | 10 | Yes | Yes | Yes | basically correct |
|  | 11 | Yes | / | Yes | Yes |
|  | 12 | / | Yes | Yes | Yes |
|  | 13 | / | Yes | Yes | Yes |
|  | 14 | Yes | Yes | Yes | Yes |
| Graduate students | 15 | Yes | Yes | Yes | Yes |
|  | 16 | Yes | / | Yes | Yes |
|  | 17 | Yes | Yes | Yes | Yes |
|  | 18 | Yes | Yes | Yes | Yes |
|  | 19 | / | Yes | Yes | Yes |
|  | 20 | Yes | Yes | Yes | Yes |
|  | 21 | Yes | Yes | Yes | Yes |
|  | 22 | / | Yes | Yes | Yes |
|  | 23 | Yes | / | Yes | Yes |
|  | 24 | Yes | Yes | Yes | Yes |

**Table S7.** **Number of students in excellent, good, moderate, pass and poor.**

| Report | Group | 90-100  (excellent) | 80-89  (good) | 70-79  (moderate) | 60-69  (pass) | ＜60  (poor) |
| --- | --- | --- | --- | --- | --- | --- |
| Analysis of ICE*Sma*M1 identification | Undergraduate students | 15 | 20 | 6 | 1 | 0 |
|  | Graduate students | 14 | 13 | 3 | 0 | 0 |
| Description of ICE*Sma*M1 phylogenetic relationship | Undergraduate students | 19 | 16 | 6 | 1 | 0 |
|  | Graduate students | 19 | 9 | 2 | 0 | 0 |
| Description of the ICE*Sma*M1 conjugation transfer and verification | Undergraduate students | 14 | 23 | 3 | 2 | 0 |
|  | Graduate students | 12 | 16 | 2 | 0 | 0 |
| Explanations for the ICE*Sma*M1 horizontal transfer | Undergraduate students | 16 | 14 | 10 | 2 | 0 |
|  | Graduate students | 15 | 14 | 1 | 0 | 0 |


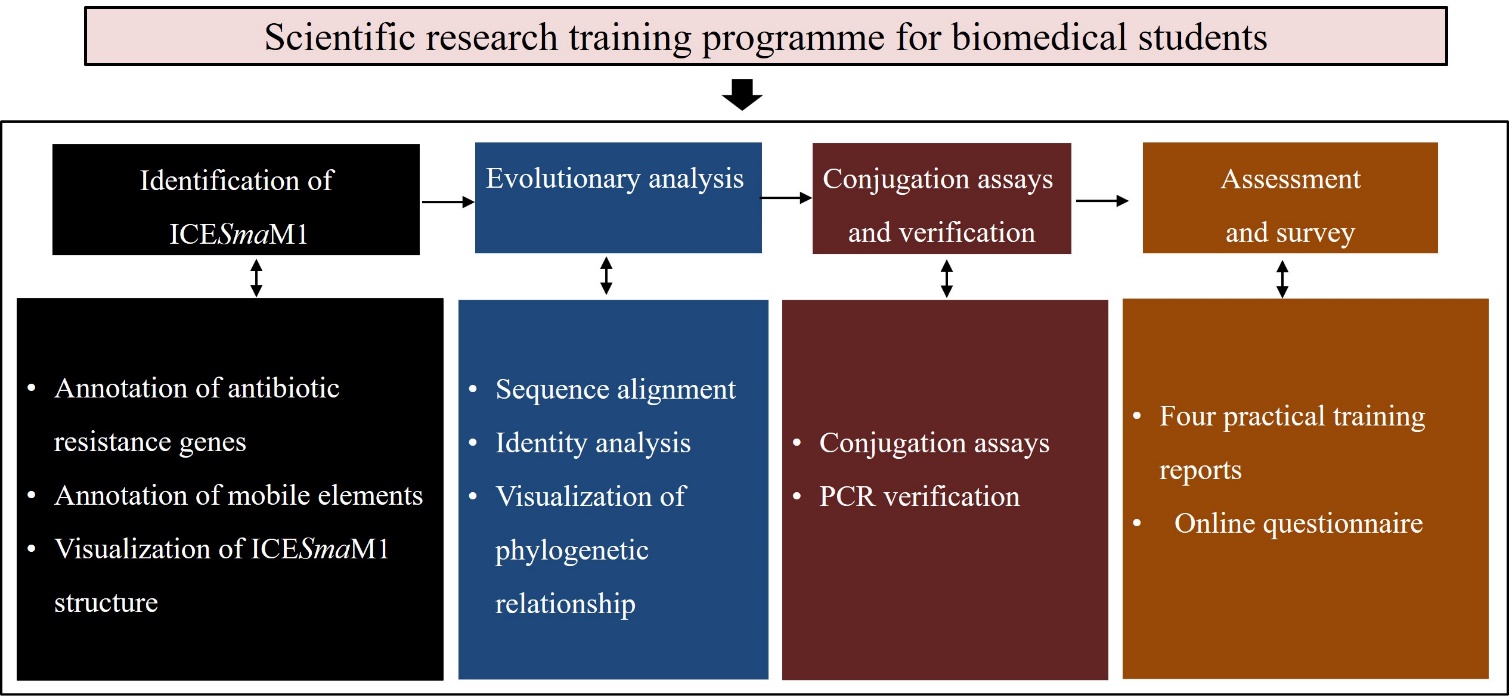


**Figure S1.** Design and framework of the scientific research training programme.
